# A comparative experimental investigation of dynamic viscosity of ZrO_2_/DW and SiC/DW nanofluids: Characterization, rheological behavior, and development of new correlation

**DOI:** 10.1016/j.heliyon.2023.e21113

**Published:** 2023-10-17

**Authors:** Ahmed M. Ajeena, Istvan Farkas, Piroska Víg

**Affiliations:** a Doctoral School of Mechanical Engineering, Hungarian University of Agriculture and Life Sciences, Szent István campus, Páter K. u. 1, Gödöllő H-2100, Hungary; b Institute of Technology, Hungarian University of Agriculture and Life Sciences, Szent István campus, Páter K. u. 1, Gödöllő H-2100, Hungary; c Institute of Mathematics and Basic Science, Hungarian University of Agriculture and Life Sciences, Szent István campus, Páter K. u. 1, Gödöllő H-2100, Hungary; d Department of Mechanical Engineering, Faculty of Engineering, University of Kufa, Iraq

**Keywords:** Dynamic viscosity, Nanofluids, Rheological behavior, Characterization, ZrO_2_ nanoparticles, SiC nanoparticles, Correlation, Experimental

## Abstract

In general, A nanofluid is a substance in which solids and fluids are mixed. The nano-powder of zirconium oxide (ZrO_2_) and silicon carbide (SiC) was dispersed into the distilled water (DW) using the widely adopted two-step technique. A Brookfield viscometer was used to measure the viscosity of the nanoparticles of ZrO_2_/DW and SiC/DW, where the temperature ranged between 20 and 60 °C and different solid volume fractions of 0.025, 0.05, 0.075, and 0.1 % were used. An examination of the mono nanofluids of ZrO_2_/DW and SiC/DW was conducted to assess their rheological behaviour. The findings of the experiments revealed that the Newtonian behaviour did not change when the nano-powder was added. Increasing the solid volume fraction of the nanoparticles and lowering the temperature resulted in the sample's dynamic viscosity being augmented. Hence, as the temperature rose, nanoparticles had a more observable impact on the viscosity. Furthermore, the findings showed that the increase in the ZrO_2_/DW nanofluid's viscosity peaked at 226.3 %, whereas for the SiC/DW nanofluid, it was 110.5 %. Additionally, according to the results of the experiments, new correlations capable of predicting the investigated nanofluids' viscosity in relation to solid concentration and temperature has been suggested. The study's results could motivate expanded utilization of nanofluids by researchers working on energy applications.

## 1 Introduction

In recent years, scientific researchers have been seeking novel ways of enhancing the heat transfer ability of different systems designed to move heat. In this regard, nanofluids are regarded as being a particularly efficient method of enhancing different thermal systems’ thermal performance. Nanofluids constitute standard fluids (e.g., engine oil, water, and ethylene glycol) in which ultrafine particles are suspended. Such novel fluids have recently been the subject of increased focus among scientists due to their capability to facilitate perceptible transfer of heat compared with standard fluids. There is widespread agreement that a characteristic of novel nanofluids that has particular significance when applied in engineering and industrial contexts is their level of viscosity. consequently, a significant body of literature has described the importance of viscosity in the implementation of nanofluids [1,2]. However, considering that when nanoparticles are added to fluids, their dynamic viscosity is increased, this enhancement also has a direct impact on the velocity field, friction forces and the ultimate ability of different kinds of thermal systems to transfer heat. Nanofluids can be differentiated from their base fluids with respect to their thermophysical characteristics, specifically thermal conductivity, and viscosity, which have received the most attention. Investigating fluid flow systems that incorporate nanofluids requires an understanding of the viscosity of nanofluids. In general, the addition of nanoparticles into the base fluid causes its viscosity to increase [[3], [4], [5]] [[3], [4], [5]] [[3], [4], [5]].

Today, as technology is developed across various fields of industry, it is unavoidable that industrial applications will be used that have increased power and thermal load; resultantly, state-of-the-art systems designed to optimise cooling are needed to a greater extent. Generally, the optimization of thermal systems is performed by enlarging the surface area, which causes the size and volume of machinery to be increased; hence, it is necessary to develop systems of cooling with increased efficiency to resolve this issue [[6], [7], [8]] [[6], [7], [8]] [[6], [7], [8]]. As standard fluids have reduced thermal conductivity, the rate of heat transferred is reduced, which is regarded as being highly problematic. A summarized review of the previous studies on the dynamic viscosity of nanofluids is presented in Table 1.

**Table 1:** A summary of studies for the dynamic viscosity of nanofluids.

Researchers	Nanoparticle	Base fluid	Temperature (^◦^C)	Solid volume fraction (%)
Sundar et al. [9]	Fe_3_O_4_	EG-water	**0–**50	**0**–**1**
Yu et al. [10]	ZnO	EG	0.2–5	0.2–5
Khedkar et al. [11]	TiO_2_	EG	10–50	1–7
Baratpour et al. [12]	SWCNT	SWCNT	30–60	0–0.1
Elias et al. [13]	Al_2_O_3_	EG: water	10–50	0–1
Said et al. [14]	Al_2_O_3_	EG	20–50	0.05–0.1
Hemmat Esfe et al. [15]	MgO	Water	24–60	<1
Hemmat Esfe et al. [16]	MWCNT	Water	25–55	0.05–1
Duan [17]	Graphite	water	25–60	1–4
Tseng and Lin [18]	TiO_2_	Water	25–50	5–12
Kulkarni et al. [19]	CuO	Water	5–50	5–15
Wang et al. [20]	Fe_3_O_4_	Water	20–60	0.5–5
Sabiha et al. [21]	SWCNT	Water	20–60	0.05–0.25

Throughout the last decade, several subfields within nanoscience have achieved notable progress and innovations paralleling the developments made by the higher scientific community. Zirconium (Zr) has unique characteristics that make it very useful for several markets. Zirconium nanoparticles have several uses as nanocatalysts, nanosensors, adsorbents, and various other forms of nanomaterials. In addition, zirconium considers benefits in many applications including chemical industry, medical devices, optoelectronics, energy storage, fuel cells, water purification, and military industry. Silicon (Si) offers an important part as an element in a diverse range of nanomaterial heterostructures, with a wide range of interesting characteristics. The utilization of their absorptive characteristics provides them with importance in the field of papermaking. It can operate as a cohesive element in the production of plastics and concrete. Furthermore, they are non-toxic and stable materials having many applications in biomedicine, light-emitting applications, energy, electronic field, and photocatalysts. Nanoparticles of (Si) and (Zr) are widely available, and their increased specific surface area and hydrophilic properties suggest they could find widespread application in industrial settings. Various approaches have been developed for this to be accomplished, and a recent technique that has attracted particular interest involves nanofluids. Certainly, the development of nanofluids represents significant progress in the area of heat transfer, and the pioneer in this field was Choi et al. [22]. Due to the fact that the size of the substances dispersed throughout the base fluids are measured in nanometres in comparison to additives whose measurements are millimetres or micrometres, such colloids offer multiple advantages including reduced erosion and pressure drop, they are more thermally conductive and stable, and the fact that the viscosity is not increased to the same extent means that less power is required to pump them [[23], [24], [25]]. The advantages related to the utilization of zirconium oxide and silicon carbide nanoparticles are explained as follows.

• The addition of ZrO_2_ and SiC nanoparticles into base fluids enhances the thermal physical properties of the base fluid due to the superior qualities exhibited by the nanoparticles compared to the base fluid.
• Zirconium dioxide (ZrO_2_) and silicon carbide (SiC) nanoparticles can be widely obtained and easy to synthesize.
• The utilization of ZrO_2_ and SiC nanoparticles has favorable economic sustainability due to their low cost.
• These nanoparticles have better stability in water as compared to several other types of nanoparticles.
• No evidence of toxicity or flammability was discovered during the utilization of these nanoparticles, indicating their environmentally friendly nature.
• ZrO_2_/DW and SiC/DW nanofluid have shown significant thermophysical properties for a wide range of applications, including heat exchangers, renewable energy, electronics cooling, and thermal storage.

Literature is enriched with studies on the viscosity of nanofluids. In this context, Bahrami et al. [26] conducted an experimental study to comprehend the changes in the viscosity of the hybrid nanofluid with fluctuations in temperature and solid volume fraction. The nanofluid was composed of water/ethylene-glycol base fluid with dispersed silicon dioxide/carbon nanotubes. It became evident that nanofluid showed greater viscosity at greater solid volume fractions and showed lower viscosity at increasing temperatures. They also examined the effects of different shear rates on the behavior of nanofluid and observed the Newtonian behavior of the base fluid and the non-Newtonian behavior of nanofluid at all concentrations. The water-based nanofluids were considered by Toghraie et al. [27] in their research; they used nanofluids with magnetic nanoparticles of Fe_3_O_4_ and examined their performance in heating and cooling systems. They revealed that the nanofluid depicted a considerable rise of 129.7 % in its viscosity when a 3 % volume of nanoparticles was incorporated into the base fluid. They also put forward an equation to determine the viscosity of water-based magnetic nanofluid Fe_3_O_4_. The value evaluated from the equation was almost the same as that evaluated practically. Another notable work was done by Etaig et al. [28] who used nanofluids for the evaluation of the new effective viscosity model. The model simulations indicated a rise in the effective viscosity of the nanofluid at higher concentrations of nanoparticles. They found an increase in the nanofluid's viscosity at higher concentrations of the nanoparticles in a nanofluid. Considering temperature changes, the viscosity increased as the temperature lowered. Hemmat Esfe [29]experimented to determine the viscosity of the MWCNT (25 %)/MgO (75 %)/10W40 NF nanofluid under the subsequently given conditions; solid volume fraction between 0 and 1 %, temperature settings between 5 and 55 °C and shear rate between 6665 and 11997 s^−1^. He discovered that nanofluid depicted greater dynamic viscosity with a rise in solid volume fraction and also depicted a decline in dynamic viscosity with a reduction of temperature. They noted the highest value of viscosity equalling 26 % at temperature settings of 45 °C and nanoparticle concentration of 1 %, and the lowest viscosity of 5 % at a temperature of 15 °C and nanoparticle concentration of 0.05 %. Afrand et al. [30]. demonstrated that when Fe_3_O_4_ nanoparticles are dispersed into water, the Newtonian behaviour of the base fluid remained constant. Additionally, they found that decreasing the temperature and increasing the volume fraction resulted in the amplification of the nanofluid viscosity. Pastoriza-Gallego et al. [31]. conducted a study in which the effects of the size of particles, solid weight fraction and temperature on CuO/water nanofluids' viscosity were investigated. Weight fractions with a maximum of 10 % and a temperate range between 10 °C and 50 °C were used in the experiments. According to their findings, as the solid weight fraction increased, the nanofluids' viscosity increased in parallel, whereas it decreased as the temperature and nanoparticle size increased. In the study of Azmi et al. [32], the viscosity of SiO2/water nanofluid with solid volume fractions varying between 0.5 % and 4 % as well as a temperature of 30 °C was measured. Their results demonstrated that the increase in viscosity reached 50 % with a nanoparticle volume fraction of 4 %. In the study of Duangthongsuk and Wongwises [33], the viscosity and temperature-dependent thermal conductivity TiO_2_–water nanofluids was measured. They demonstrated that an increase in the concentration of particles caused both variables to increase to a level that exceeded that of the base fluids. Non-Newtonian nanofluids' rheological properties were analysed in the study of Hojjat et al. [34]. They reached the conclusion that temperature and the concentration of particles influence the rheological attributes of such fluids. The thermal conductivity and viscosity of nanofluids in which carbon nanotubes with multiple walls were contained were studied by Phuoc et al. [35].The findings showed that increases in the thermal conductivity were not related to the viscosity of the base fluid, suggesting that enhanced thermal conductivity is not significantly influenced by the particle velocity. A nanofluid based on ethylene glycol containing particles of SiO_2_ was investigated in the study of Rudyak et al. [36] for the purpose measuring the viscosity coefficient. They reported that a nanofluid's viscosity coefficient is highly dependent on temperature. Sadri et al. [37] demonstrated the ability of carbon nanotubes (CNTs) to enhance the thermal performance of standard working fluids. Their results showed that sonication time is a factor that influences the thermal conductivity and viscosity of such nanofluids. In the research of Li et al. [38], ZnO nanofluids based on ethylene-glycol were analysed in terms of their viscosity and thermal conductivity. They reached the conclusion that as expected, an increased concentration of ZnO nanoparticles caused the nanofluid's viscosity to increase, whereas it decreased as the temperature rose. Jarahnejad et al. [39] explored how the dynamic viscosity of nanofluids based on water that contained nanoparticles of titania (TiO_2_) and alumina (Al_2_O_3_) were effected by factors such as temperature as well as nanoparticle size and concentration. Their findings indicated that in general, the viscosity of the nanofluids that contained nanoparticles of TiO_2_ nanoparticles was greater compared to those containing Al_2_O_3_ when the loading was the same. Abbasi et al. [40] investigated the effects of temperature and concentration on the flow behaviour and viscosity of nanofluids containing TiO_2_ nanoparticles, pristine MWCNTs, oxidised MWCNTs, and decorated MWCNTs were impacted by concentration and temperature in terms of the viscosity and flow characteristics. Their findings revealed that nanofluids' properties are dependent on concentration and temperature, while a decrease in the viscosity of the produced nanofluids was observed as the temperature rose and the concentration decreased. According to data from previous studies, a novel correlation was proposed by Meybodi et al. [41] on the basis of an exhaustive database of the viscosity data of Al_2_O_3_, TiO_2_, SiO_2_, and CuO nanofluids based on water. Their outcomes indicated that the developed correlation generated predictions that higher levels of agreement than values produced from experiments compared to past models, particularly in cases involving increased values of temperature, volumetric concentration and viscosity. Hemmat Esfe et al. [42] investigated how nanoparticle load and temperature impacted the viscosity of a nanofluid containing CuO, where the base fluid consisted of ethylene glycol. The results of their investigation indicated that the nanofluid relative viscosity value for the nanofluid containing 1.5 vol% CuO nanoparticles at 50 °C peaked at 82.46 %. Additionally, the developed a model design to predict the viscosity of a nanofluid and demonstrated that the data obtained from experimentation only deviated from those generated by the model by approximately 4 %. The results of the experiments also showed that a significant decrease in the dynamic viscosity occurred as the temperature of the nanofluid increased. Additionally, Murshed et al. [43] conducted measurements on nanofluids containing TiO_2_ and Al_2_O_3_ to determine their thermal conductivity and dynamic viscosity. They reached the conclusion that the nanofluids had significantly increased values for these parameters compared to the unaltered base fluids. Furthermore, they detected significant increases in the dynamic viscosity and thermal conductivity values when the volume fraction of the nanoparticles increased from 0.01 to 0.05 vol%. Chandrasekar et al. [44] investigated the effective viscosity of a nanofluid of Al_2_O_3_/H_2_O nanofluid whose nominal diameter was 43 nm at various volume fractions between 0.33 % and 5 % experimentally and theoretically. Preparation of the nanofluid involved the synthesis of particles of Al_2_O_3_ via a method of chemical precipitation facilitated by microwaves. Subsequently, a sonicator was used to disperse the particles in distilled water. An increase in the nanofluids' viscosity was observed proportional to the volume fraction of the nanoparticle. Conventional models have been designed that are capable of predicting nanofluid viscosity, which largely agreed with the results produced the experiments. In the study of Yiamsawas et al. [45], a nanofluid comprised of Al_2_O_3_ and water was evaluated to determine its viscosity when both the particle concentration and temperature were increased. The nanoparticles' volume fraction ranged between 1 % and 8 % as the temperature increased from 15 °C to 60 °C. The concluded that as the temperature rises, the viscosity is lowered. Jeong et al. [46] conducted experiments to investigate the viscosity of nanofluids containing ZnO whose nanoparticles were largely shaped in the form of rectangles or sphered with range of nanoparticle volume fractions between 0.05 and 5.0 vol%. The findings showed that as the volume concentration increased, the nanofluids' viscosity increased proportionally from 5.3 % to 68.6 % in comparison to water, which comprised the base fluid.

According to prior researches, the conclusion can be drawn that a nanofluid's viscosity can be directly influenced by multiple factors including temperature, methods of suspension, solid volume fraction, the shape of the particle, surfactant, kind of nanoparticles and the base fluid. The viscosity of nanofluids has been the focus of numerous studies, which can be referenced to improve the comprehension of nanofluids' viscosity characteristics. In order for nanofluids' rheological behaviour to be researched, it is firstly necessary to determine their viscous behaviour from Newtonian and non-Newtonian perspectives. The findings of certain researchers have indicated the base fluid's rheological behaviour is not changed when nanoparticles are added.

In the present study, the preparation of various samples of ZrO_2_/DW and SiC/DW mono nanofluids was conducted utilising the two-stage approach for the purpose of investigating the rheological behaviour of the nanofluids, while the XRD pattern, TEM, EDX, FESEM, DLS, UV–vis, and Zeta potential methods were used to measure its structural characteristics and stability. Measurement of the investigated nanofluids’ viscosity was performed in various solid concentrations (between 0.025 % and 0.1 %) and temperatures (between 20 °C and 60 °C). Furthermore, according to data obtained from the experiments, new correlations for predicting the dynamic viscosity of ZrO_2_/DW and SiC/DW nanofluids with respect to solid concentration and temperature have been suggested. The study also highlighted the significance of comprehending the nanofluid viscosity to get better insight into the working of fluid flow systems. In short, the incorporation of nanoparticles into the base fluid yields nanofluid with higher Viscosity.

## 2 Experimentation

### 2.1 Material

**Fig. 1:**
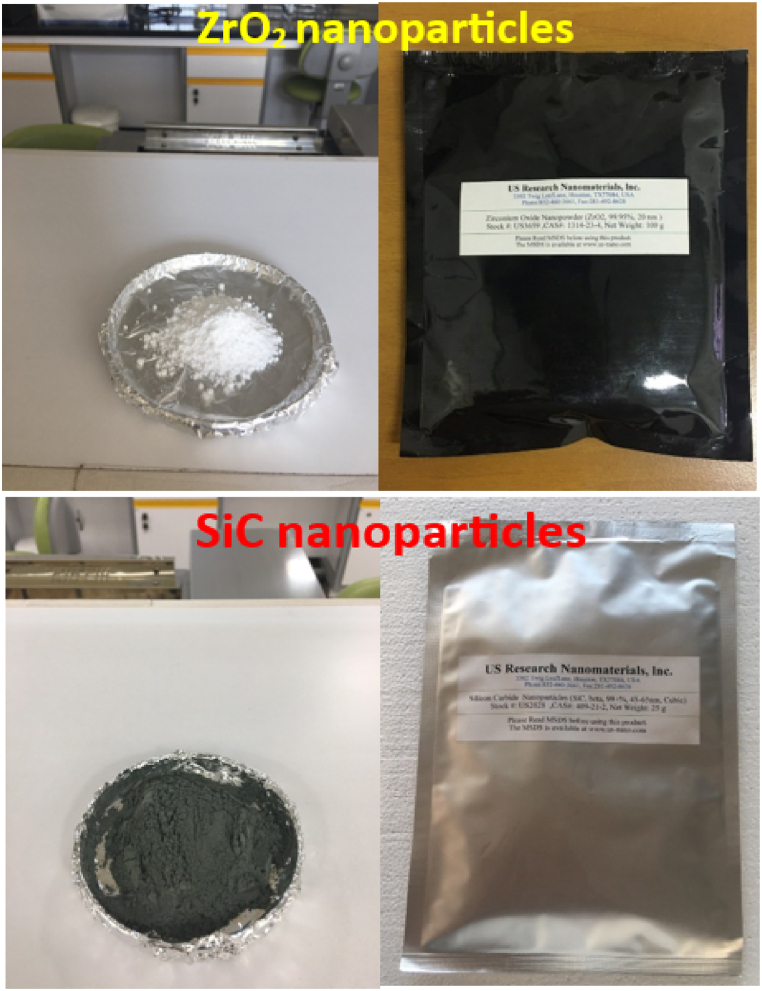
Images of zirconium oxide and silicon carbide nanoparticles.

In the present research, nanofluids for ZrO_2_/DW and SiC/DW samples at volume fractions of 0.025, 0.05, 0.075, and 0.1 % were created by dispersing nanoparticles of ZrO_2_ and SiC into the distilled water. Table 2 present summary of the thermophysical characteristics of nanoparticles of ZrO_2_ and SiC, respectively, which were sourced from US Research Nanomaterials. The images of zirconium oxide and silicon carbide nanoparticles are shown in Fig. 1.

**Table 2:** Physical and chemical properties of zirconium oxide and silicon carbide nanoparticles.

Properties	Zirconium Oxide	Silicon Carbide
Chemical formula	ZrO_2_	SiC
Purity (%)	99.95	99
Color	white	Grayish white
Morphology	near spherical	cubic
Specific Surface Area (SSA) (m2/g)	30–60	40–80
Actual particle size (APS) (nm)	20	45–65
Stock code	US3659	US2028
CAS No.	1314-23-4	409-21-2
Density (ρ) (g/cm3)	5.89	3.216
Specific heat (J/kg. K)	455	680
Thermal conductivity (W/m·k)	2.7	370

### 2.2 Nanofluid preparation

**Fig. 2:**
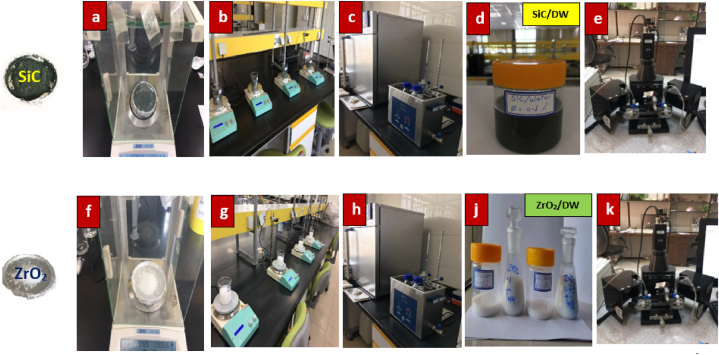
Preparation process of ZrO_2_/DW and SiC/DW nanofluids:(a, f) Digital scale, (b, g) Magnetic stirrers, (c, h) Ultrasonic, (d) Silicon Carbide nanofluid, (j) Zirconium Oxide nanofluid, and (e, k) Zeta potential analyzer.

A two-step approach was used to obtain samples of ZrO_2_/DW and SiC/DW nanofluids. Subsequent to weighting, nanoparticles of ZrO_2_ and SiC at the intended solid volume fraction (0.025, 0.05, 0.075, and 0.1 %) were added to purified water. It was necessary to firstly calculate the necessary mass amounts before the various nanofluid fractions could be prepared. For the purpose of calculating the nanoparticle and base fluid values and the respective amounts needed to prepare various volume fractions, Eq. (1) was employed. A digital scale (A&D WEIGHING, GE-320, USA) with high sensitivity was used for measuring the materials' weight. When the experiment started, computation of the mass amounts was performed in terms of the nanoparticles’ density. Subsequent to the addition of nanoparticles to the water, the mixture was stirred using a magnetic stirrer (HS-12, HU) for approximately 1 h. Afterward, suspensions were entered into an ultrasonic processor (PS 30A 6L, Germany) for the process of breaking down the agglomeration among the particles as well as preventing sedimentation, ensuring the particles were uniformly dispersed and the suspension was stable; this process lasted approximately 3–4 h. Fig. 2 shows preparation process of ZrO_2_/DW and SiC/DW nanofluids.

(1) $$\varphi =\left[\frac{{\left(\frac{w}{\rho }\right)}_{nanoparticles}}{{\left(\frac{w}{\rho }\right)}_{\text{nanoparticles}}+{\left(\frac{w}{\rho }\right)}_{\text{DW}}}\right]\times 100$$

### 2.3 Dynamic light scattering (DLS) analysis

**Fig. 3:**
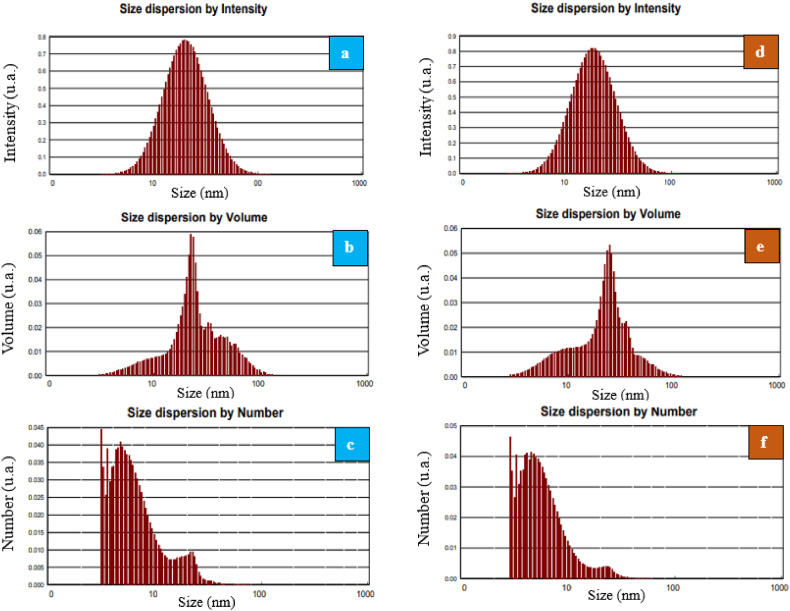
DLS analysis for: (a, b, c) SiC/DW nanofluid between intensity, volume, and number, respectively and (d, e, f) ZrO_2_/DW nanofluid between intensity, volume, and number, respectively.

After being dispersed into liquid phase, the synthesised nanoparticles’ hydrodynamic size distribution was measured with dynamic light scattering (DLS). The respective particle size distributions for the nanoparticles of silicon carbide and zirconium oxide according to number, volume, and intensity are shown in Fig. 3. As a result of the data analysis, according to the DLS

measurements, the nanoparticles of SiC and ZrO_2_ had respective sizes is less than 100 nm, where the distribution of sizes was limited.

### 2.4 Measuring dynamic viscosity

**Fig. 4:**
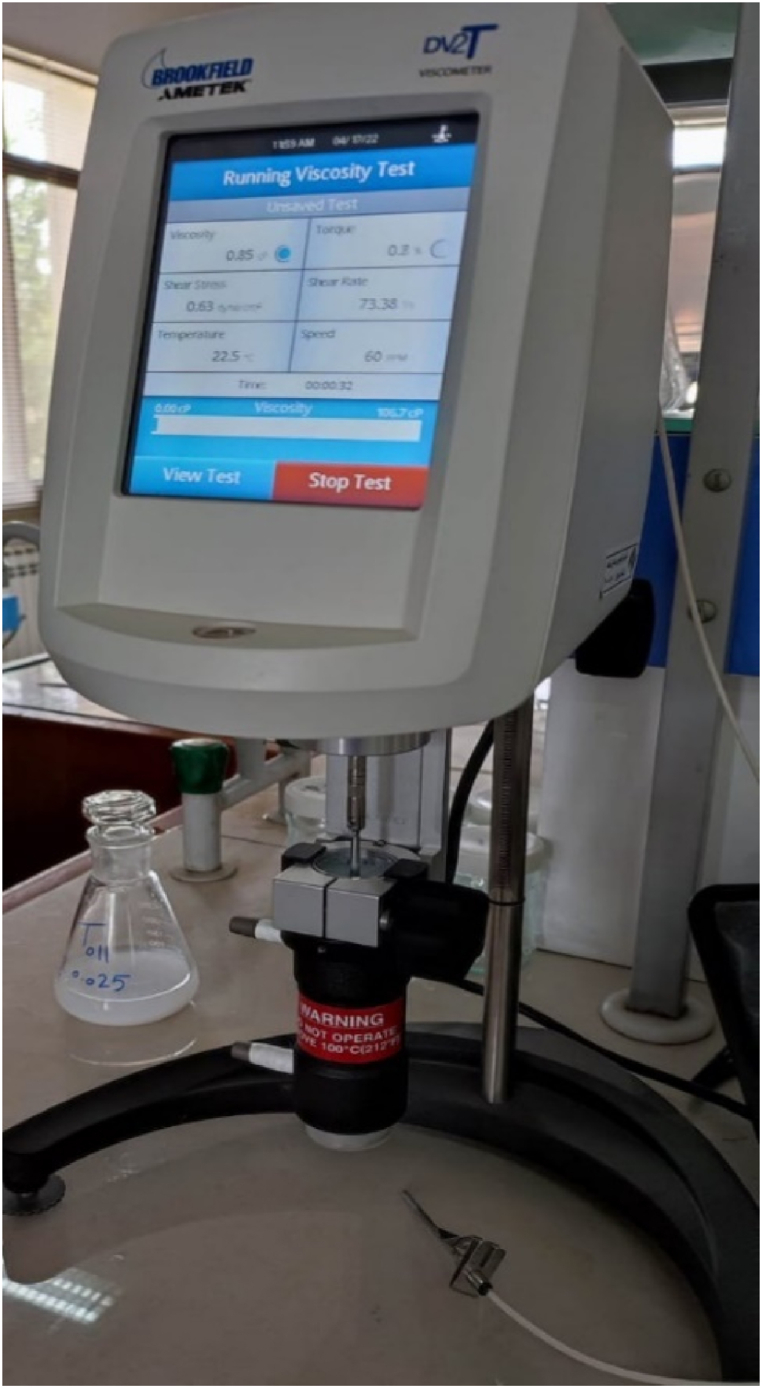
Brookfield rotational viscometer (DV2TRVTBG).

In the current study, a Brookfield DV2TRVTBG rotational viscometer was used for measuring the ZrO_2_/DW and SiC/DW nanofluids' viscosity as well as for identifying their rheological characterization when influenced by varying parameters. Such viscometers function by taking measurements of fluid's resistance caused by the torque from the spindle placed into the fluid. Additionally, to ensure the temperature remained stable as well as to determine how nanofluids' viscosity is impacted by temperature, a temperature bath with significant accuracy was linked with the viscometer. Hence, this facilitated the process of conducting analysis of the nanofluids' rheological properties and viscosity according to the effects of shear rate and temperature at varying concentrations. Therefore, via the connection between the viscometer and temperature bath, the parameters of the experiment were fixed such that the temperature ranged between 20 and 60 °C to measure the nanofluids' dynamic viscosity at different solid volume fractions. All measurements were performed using a ULA spindle that was a small for sampling adapter system. Furthermore, calibration of the viscometer was performed prior to taking measurements using water as the base fluid at an ambient temperature. An image of the Brookfield viscometer is depicted in Fig. 4.

## 3 Results and discussion

### 3.1 Characterization

**Fig. 5:**
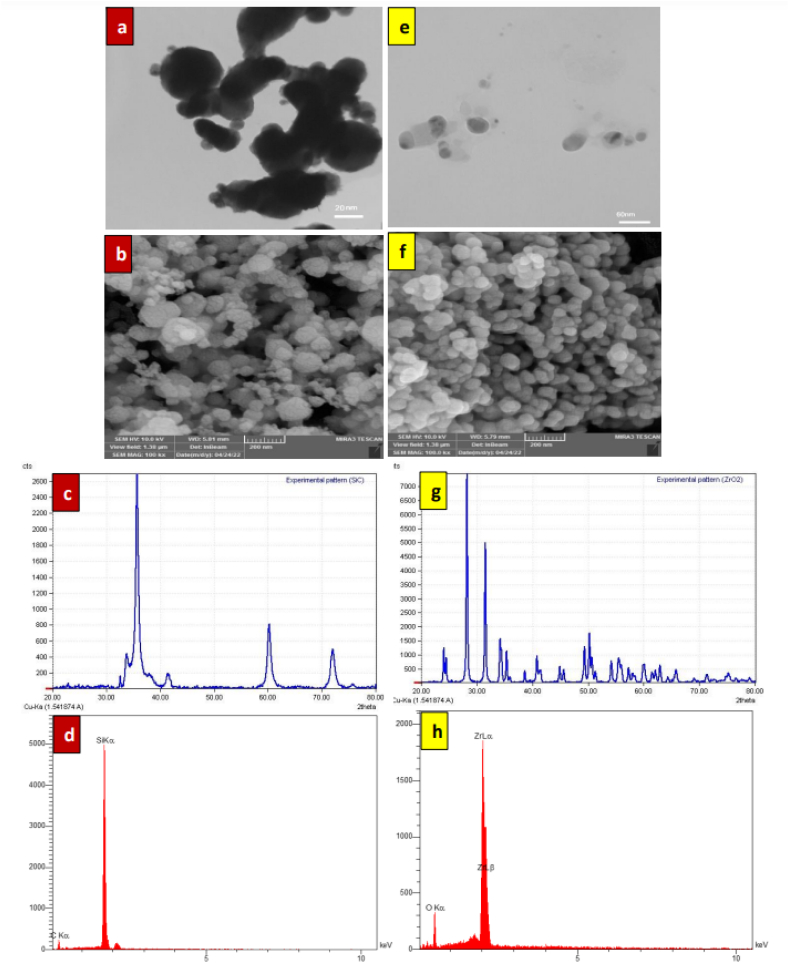
TEM, FESEM, XRD, and EDX techniques for (a, b, c, d) SiC nanoparticles and (f, e, g, h) ZrO_2_ nanoparticles.

The structural properties of SiC and ZrO_2_ nanoparticles are measured by applying X-ray diffraction. The XRD instrument is used to obtain the angle of the incident (theta), and angle of reflection to maintain a fixed focusing distance by modifying the relative places of the reflected X-ray, sample surface and incident X-ray. Therefore, there will be an angle of 2θ between the reflected and incident X-ray. In contrast, the degree of crystallinity of the exact plane is shown by the peak intensity since crystallinity is a relative term and not fixed. X-ray diffraction spectra of SiC and ZrO_2_ are shown in Fig. 5-c and 5-g, respectively. There is an intense peak (diffraction peaks) at 2θ = 35.78° is attributed to silicon carbide and 2θ = 28.29° for zirconium oxide. Low-intensity peaks can also be observed in the XRD plot.

The samples film of SiC and ZrO_2_ dry nanoparticles are subjected to a ray of high-energy electrons for accurate measurement of nanoparticle size and shape. The electrons move through the sample and produce an image on the screen. The presence of near spherical shaped for ZrO_2_ and cubic shape for SiC nanoparticles are confirmed by the TEM images. The TEM images also indicate nanoparticles to be characterized with a 20 nm average size for ZrO_2_ (Fig. 5-e) and rang size between 45 and 65 nm for SiC nanoparticles (Fig. 5-a).

Field emission scanning electron microscopy (FESEM) is one of the most suitable methods that can be used to discover the morphological properties of nanoparticles. FESEM images with the magnification scale of 200 nm can be seen in Fig. 5-b and 5-f that as expected, there is a layered structure of silicon carbide and zirconium oxide nanoparticles with nano-size. silicon carbide and zirconium oxide nanoparticles are enabled by the planar surface and interconnected surface to generate conduction paths by generating networks. In addition, a spherical shape of ZrO_2_ nanoparticles is shown in Fig. 5-f with a size 20 nm. Also, Fig. 5 b show morphological cubic of SiC nanoparticles with size between 45 and 65 nm. Particle sizes at the nanoscale were stressed by both FESEM images. Fig. 5-d and 5-h shows the chemical compositions of SiC and ZrO_2_, respectively, the determination of which was made through EDX analysis. The results indicated that 53.26 % carbon, and 46.74 % silicon were present in the SiC while 67.88 % zirconium, 32.12 % oxygen were detected in ZrO_2_.

### 3.2 Stability of nanofluid

**Fig. 6:**
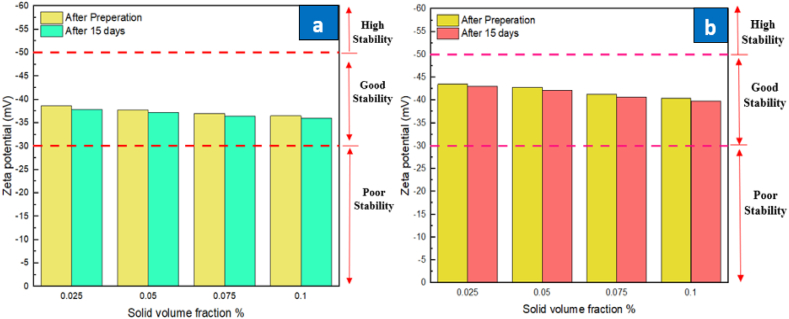
Zeta potential analysis of (a) ZrO_2_/DW and (b) SiC/DW nanofluids.

Initially, a zeta potential test is performed to check the stability of the prepared samples of Sic and ZrO_2_. Next, the samples are poured inside an ultrasonic device where it is ensured that particles are uniformly dispersed in the sample by treating it at 20 kHz frequency for 40 min. The resultant nanofluid shows higher stability. After about 15 days, the nanofluid samples with different volume concentrations (0.025 %−0.1 %) were checked for stability revealing adequate stability. Fig. 6 depicts the values of zeta potential test conducted on the given samples of SiC and ZrO_2_ nanofluids. The zeta potential test allows evaluation of the disparity in the electrical potential of the slipping layer and that observed at distance from the particle. The density of particles is directly associated with zeta potential. The surface charge on the particles results in colloid stability. The particles experience a repulsion force between them due to similarly charged loads and thus, they remain apart from each other leading to higher stability and weaker agglomeration. The results indicated stable nanofluid as evident form the values of zeta potential shown in this figure. The criteria suggest that nanofluids with zeta potential values between 30 and 50 mV are considered stable.

### 3.3 Validation of measurements

**Fig. 7:**
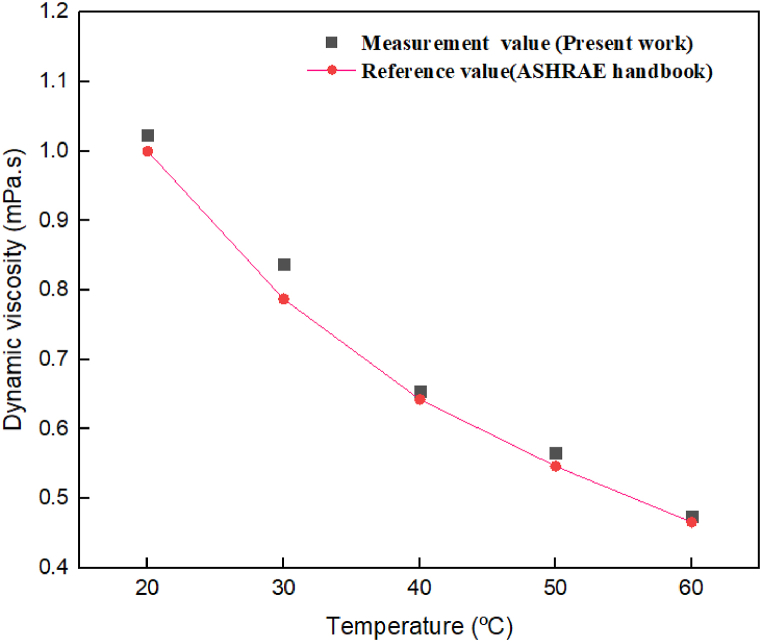
Comparison between ASHRAE data on viscosity of water and experimental outcomes at different temperatures.

Validity testing is essential in experimental investigations because of the variety of possible causes of inaccuracy. Pure water's dynamic viscosity was measured with a Brookfield rotational viscometer at different temperatures, and outcomes were compared with the values found in the ASHRAE handbook [47]. The results are displayed in Fig. 7, which indicates an acceptable level of agreement between the measured values and the reference (ASHRAE Handbook). This finding provides confirmation of the measuring instruments' high level of accuracy.

### 3.4 Rheological behavior

The Prandtl and Reynolds number of nanofluids is influenced by the viscosity. Hence, it is a critical thermophysical characteristic for convective heat transfer, lubrication and pumping power. For the purpose of evaluating the rheological properties of ZrO_2_/DW and SiC/DW nanofluids, measurements of the nanofluids' viscosity were taken at varying shear rates. A fluid's rheological behaviour is based on the given Eq. (2).

(2) τ=μγ

**Fig. 8:**
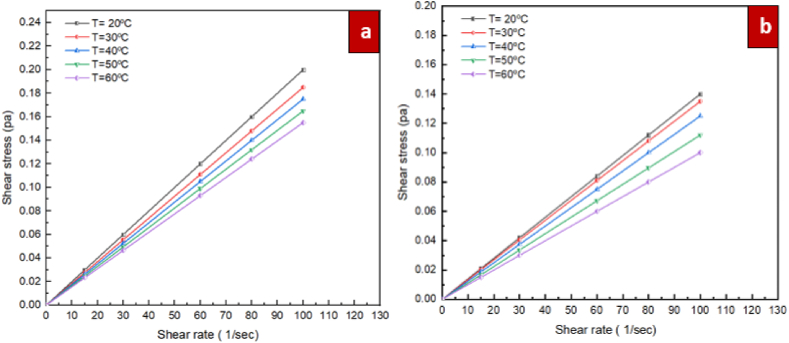
Shear stress vs. shear rate at solid volume fraction of 0.1 % for different temperatures, (a) ZrO_2_/DW and (b) SiC/DW nanofluid.

**Fig. 9:**
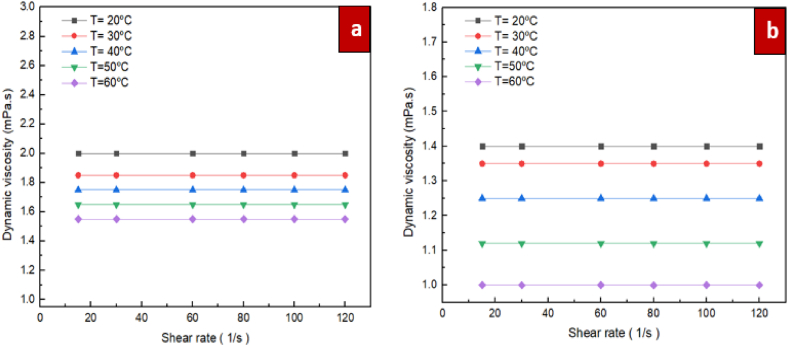
Dynamic viscosity vs. shear rate at solid volume fraction of 0.1 % for different temperatures, (a) ZrO_2_/DW and (b) SiC/DW nanofluid.

Where γ, τ, and μ denote the shear rate (1/s), dynamic viscosity (Pa·s), and shear stress (Pa), respectively. Based on this equation, a fluid is defined as being Newtonian in cases where the shear stress is a linear function of the shear rate. In Fig. 8, the shear stress is shown as a function of shear rate for mono nanofluids of ZrO_2_/DW and SiC/DW where the solid volume fraction is 0.1 % and the temperatures vary. As the changes in shear stress as result of the shear rate show a linear pattern, it can be deduced that the ZrO_2_/DW and SiC/DW nanofluids exhibit Newtonian characteristics, which supports the standard rule proposed by Venerus et al. [48]. This is a fundamental requirement for the use of nanofluid in thermal systems for applications like convection. Fig. 9 demonstrates the changes in dynamic viscosity according to the shear rate at various temperatures and volume fraction of 0.1 % for the nanofluids of ZrO_2_/DW and SiC/DW. As the dynamic viscosity remains constant with regard to the shear rate, this suggests that the nanofluids of ZrO_2_/DW and SiC/DW behave in a Newtonian manner.

### 3.5 Dynamic viscosity

The viscosity of a fluid is a thermophysical characteristic and manifests in cases were slipping among the layers of fluid happens. It is the characteristic due to which a fluid offers resistance to relative motion among molecules of the fluid. Viscosity is more evident when the layers of the fluid experience movements. The mechanism underling the creation of viscosity in fluids is van der Waals forces among molecules. When this occurs, frictional forces are formed according to the value of viscosity. The strength of the friction force rises in line with the viscosity. The addition and dispersion of nanoparticles among the molecules of the base fluid results in tighter slippage of the fluid layers. Hence, the strength of the frictional force formed among the layers of fluid within the nanofluid is enhanced.

**Fig. 10:**
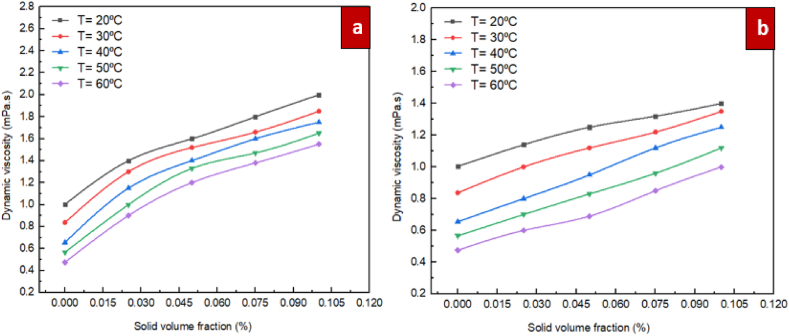
Differences of dynamic viscosity of nanofluids with solid volume fraction at different temperatures for (a) ZrO_2_/DW and (b) SiC/DW nanofluid.

In Fig. 10, the changes in dynamic viscosity according to temperature at various volume fractions are shown for the ZrO_2_/DW and SiC/DW nanofluids. As illustrated in the figure, as the temperature rises, the viscosity of the liquid is lowered. This is due to the fact that the interactions between molecules and the van der Waals forces are weakened when the temperature increases. It is an intriguing finding that when the temperature remains the same, when the solid concentration increases, the nanofluid's dynamic viscosity also increases accordingly. The rise is more discernible when temperatures are reduced as opposed to increased. Based on the findings, when the volume fractions are increased, the temperature has a more discernible impact on the viscosity of the nanofluid. Indeed, when the volume fraction is elevated, there is an increased likelihood that agglomeration will occur among solid particles within the base liquid, the bonds connecting particles break more frequently, and the changes in viscosity are more pronounced, in comparison to situations where the temperature is reduced.

**Fig. 11:**
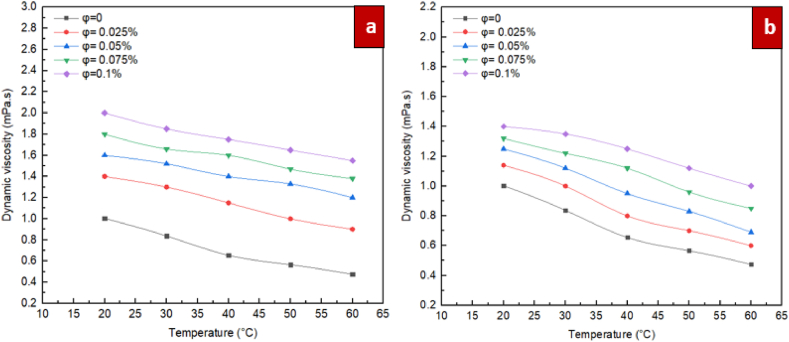
Differences of dynamic viscosity of nanofluids with temperatures at different solid volume fraction for (a) ZrO_2_/DW and (b) SiC/DW nanofluid.

In Fig. 11, the changes in dynamic viscosity for the mono nanofluids of ZrO_2_/DW and SiC/DW according to the volume fraction at various temperatures are shown. As illustrated in the figure, as the volume fraction rises, nanofluids viscosity increases accordingly. This is due to the fact that the addition of nanoparticles to the base fluid causes the molecules of the liquid and the solid particles to interact to a greater extent, thus causing the viscosity to increase. Nanoclusters with greater magnitude are formed when the proportion of solid particles within a particular volume of base liquid is increased because of the greater van der Waals forces among them. The resultant nanoclusters restrict layers of water from moving easily on each other, which causes the viscosity to rise to a large extent. Additionally, van der Waals forces improve the viscosity according to the volume when the temperature is reduced. The reason for this is that when the temperature is increased, particles are capable of overcoming van der Waals forces, which causes the rate at which the viscosity increases to decelerate as the volume fraction rises, in comparison to situations where the temperature is reduced. On the other hand, minimum increases of 39.5 % and 13.6 % were observed for the ZrO_2_/DW water and SiC/DW nanofluids, respectively, when the solid concentration was 0.025 % and temperature was 20 °C. Another remarkable finding was that when the solid concentration was 0.025 % and the temperature was set at 20, 30, 40,50,and 60 °C, the dynamic viscosity of the ZrO_2_/DW nanofluid was under 89 %, whereas it was approximately 26 % for the SiC/DW nanofluid; this shows the potential of these nanofluids for application in industrial and engineering settings due to the increased importance of pumping strength at reduced pressures. It should be noted that in the case of the ZrO_2_/DW nanofluid, the dynamic viscosity increase was maximised and minimised at 226.3 % and 39.5 %, whereas for the SiC/DW nanofluid, its maximum and minimum increase were 110.5 % and 13.6 %, which manifested at sold concentrations and temperatures of 0.025 % and 20 °C for the former, and 0.1 % and 60 °C for the latter.

### 3.6 Relative dynamic viscosity (RDV)

**Fig. 12:**
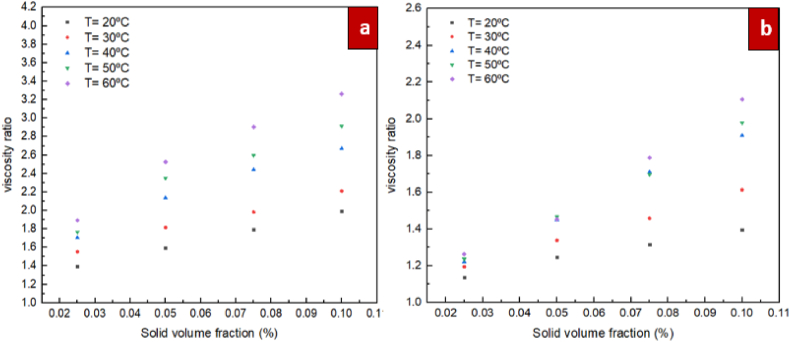
Differences of relative dynamic viscosity with solid volume fraction at different temperatures for (a) ZrO_2_/DW and (b) SiC/DW nanofluid.

**Fig. 13:**
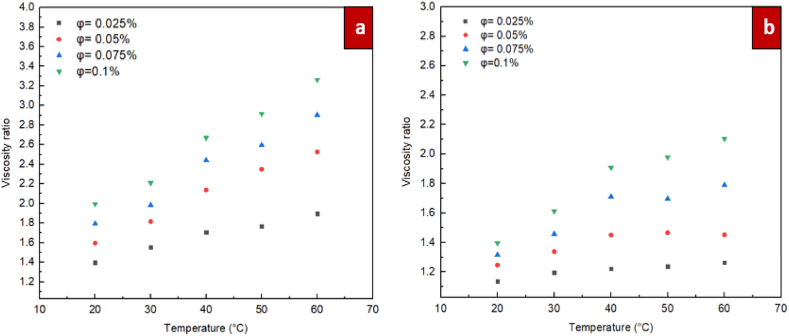
Differences of relative dynamic viscosity with temperature at different solid volume fraction for (a) ZrO_2_/DW and (b) SiC/DW nanofluid.

According to the measurements, the definition of the "relative viscosity” is based on the ratio between the dynamic viscosity of nanofluids of ZrO_2_/DW and SiC/DW and the nanofluids' dynamic viscosity (distilled water), expressed in Eq. (3). In Fig. 12, Fig. 13, the relative dynamic viscosity in relation to temperature and solid volume fraction for the nanofluids of ZrO_2_/DW and SiC/DW is shown. The figures clearly show that as the solid volume fraction is increased, the relative viscosity is increased. This pattern was also supported in past studies. As shown in Figures, the relative viscosities increased marginally as the temperature rose, and this was more observable when the solid volume fractions also increased. In the solid volume fraction range from 0.025 % to 0.05 %, and with temperature of 60 °C, the relative viscosity of ZrO_2_/DW nanofluid changed the most by 33 %, whereas for SiC/DW, it changed by 15 %.Furthermore, augmenting the particle ration with a volume fraction of 0.1 % to approximately 72 % and 67 % for the ZrO_2_/DW and SiC/DW nanofluids, respectively, caused the relative viscosity to change by the greatest amount. This result implies that volume fraction as opposed to temperature has a greater influence on nanofluids’ viscosity.

(3) $$\text{Relative}\;\text{viscosity}=\frac{\mu_{\text{nf}}}{\mu_{\text{bf}}}$$

### 3.7 Proposed correlation

When nanofluids applied in engineering, it is frequently necessary to demonstrate thermophysical characteristics as equations or by utilising mathematical programmes for the properties of the nanofluid to be used. In the current research, the fitting method along with Levenberg–Marquardt algorithm by Sigma Plot program was applied to the data obtained from the experiments, which facilitated the development of a novel model capable of predicting the dynamic viscosity of the investigated nanofluids with respect to solid concentration and temperature. New correlations for the prediction of the dynamic viscosity of nanofluids as functions of solid concentration (φ) and temperature (T) is developed through Eqs. (4), (5) for ZrO_2_/DW and SiC/DW, respectively, as shown below.

(4) $$\frac{\mu_{\text{nf}}}{\mu_{\text{bf}}}=0.99761+0.26995\times \varphi^{0.32737}-0.03587\times T^{0.89391}+0.19267\times \varphi^{0.32737}\times T^{0.89391}$$

(5) $$\frac{\mu_{\text{nf}}}{\mu_{\text{bf}}}=62.5681-16202.79566\times \varphi^{1.23096}-61.32394\times T^{0.00072885}+16172.3693\times \varphi^{1.23096}\times T^{0.00072885}$$

For the purpose of investigating the suggested correlations accuracy, it is necessary to define a specific parameter known as the Margin of Deviation, as expressed in Eq. (6).

(6) $$\mathrm{MoD}\;\left(\%\right)=\left[\frac{{\left(\frac{\mu_{\text{nf}}}{\mu_{\text{bf}}}\right)}_{\mathrm{Exp}}-{\left(\frac{\mu_{\text{nf}}}{\mu_{\text{bf}}}\right)}_{\text{Pred}}}{{\left(\frac{\mu_{\text{nf}}}{\mu_{\text{bf}}}\right)}_{\mathrm{Exp}}}\right]\times 100$$

**Fig. 14:**
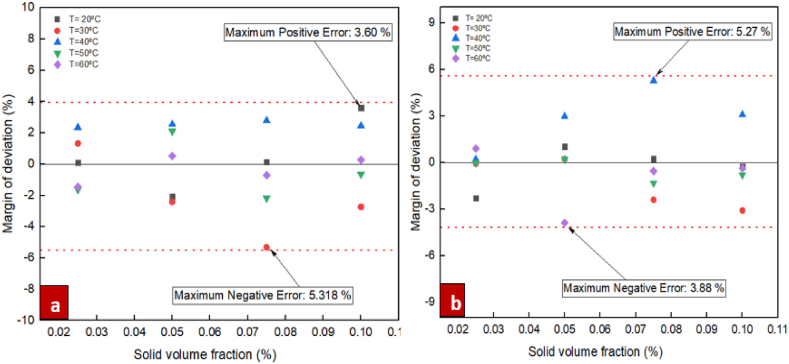
Margin of deviation for (a) ZrO_2_/DW and (b) SiC/DW nanofluid.

**Fig. 15:**
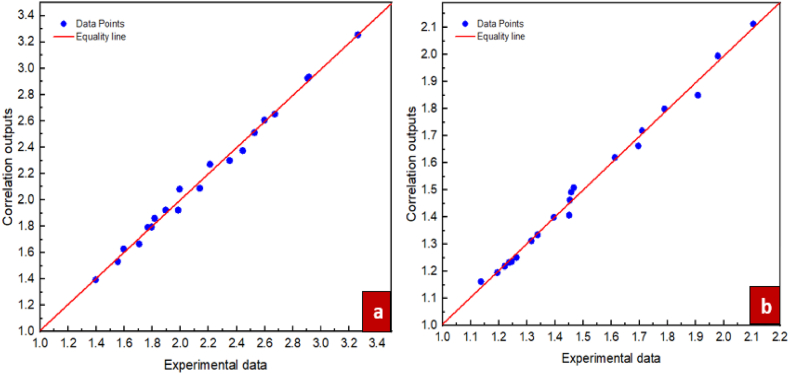
Comparison between laboratory results and outputs of proposed correlation for (a) ZrO_2_/DW and (b) SiC/DW nanofluid.

**Fig. 16:**
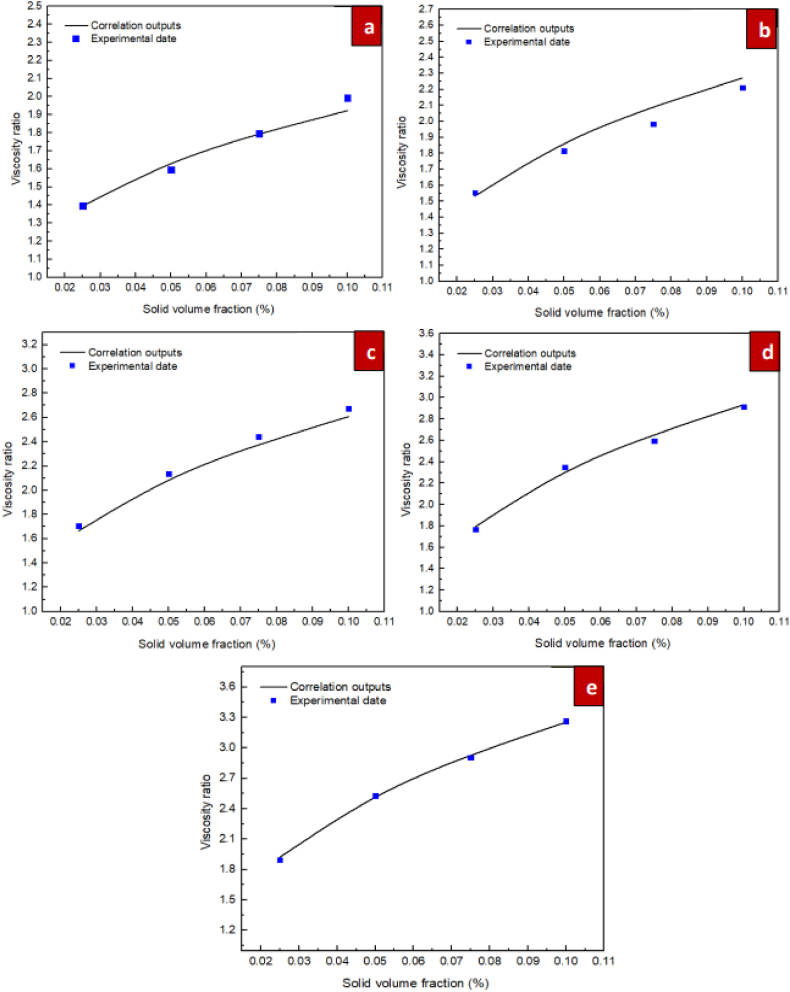
Comparisons between the laboratory results with the outcomes obtained from proposed correlation for ZrO_2_/DW in the temperatures of (a) 20 °C, (b) 30 °C, (c) 40 °C, (d) 50 °C, and (e) 60 °C.

**Fig. 17:**
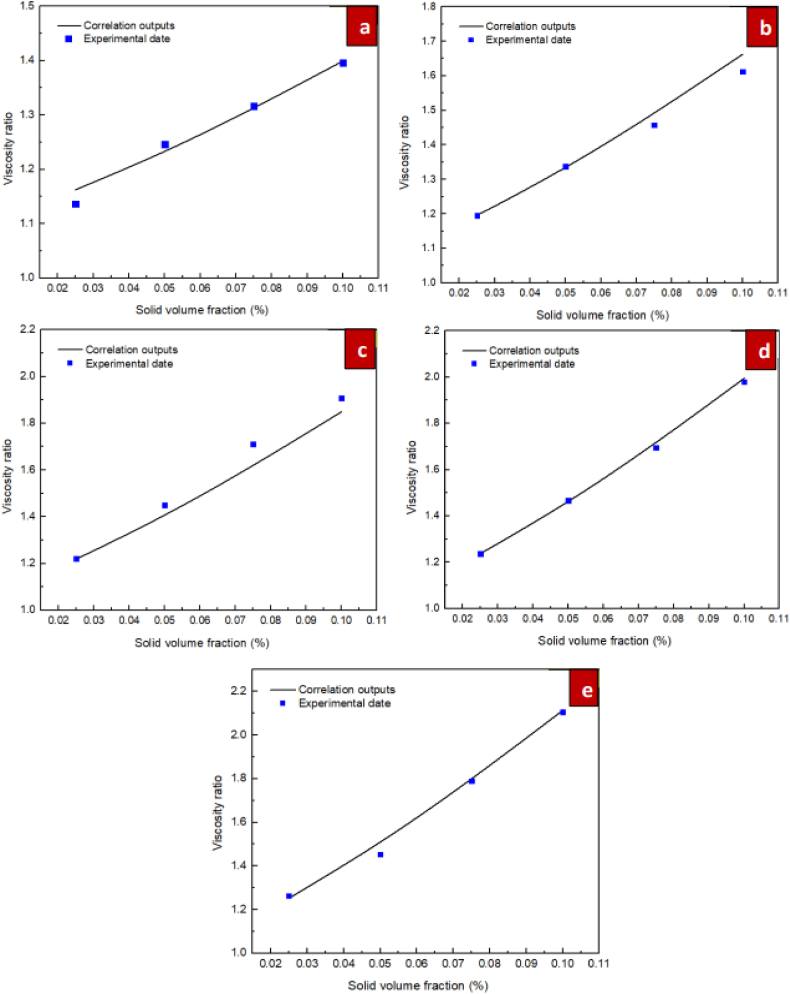
Comparisons between the laboratory results with the outcomes obtained from proposed correlation for SiC/DW in the temperatures of (a) 20 °C, (b) 30 °C, (c) 40 °C, (d) 50 °C, and (e) 60 °C.

In Eq. (6) above, the indices Exp and Pred represent experimental data and the predictions of the suggested correlations, respectively. Fig. 14 show the margin of deviation values. The different values for the ratio of dynamic viscosity acquired from the experiments and correlation are compared in figure. Additionally, this figure presents the margins of deviation calculated from Eq. (6). Moreover, for the nanofluids of ZrO_2_/DW and SiC/DW the margin of deviation peaked at 5.38 % and 5.27 %, respectively. Furthermore, Fig. 15 compare the values from the experiments and the predictions for the respective nanofluids. As these figures indicate, most points are located on or near to the bisector and it is therefore not significantly distant, which suggests that the correlation is suitably accurate. Also, the figure suggests that the data from the experiments and the correlation results have a good level of agreement. Due to the significance of the estimations of thermal conductivity being accurate, Fig. 16, Fig. 17 compare the data from the experiments and the empirical correlation results for the respective nanofluids at a range of temperatures. These figures reveal that for the majority of the measurement values, there is either overlap between the points denoting the experimental and correlation results or they deviate slightly. This trend indicates that the suggested correlation is suitably accurate.

### 3.8 Comparison between previous theoretical models and experimental outcomes

**Fig. 18:**
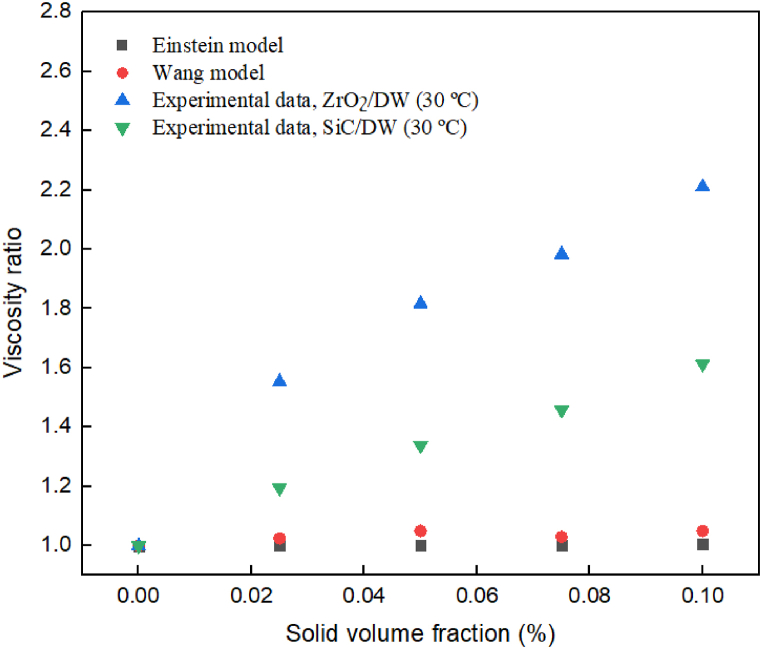
Comparison of theoretical models and experimental outcomes.

Researchers have presented several theoretical models to estimate the dynamic viscosity of nanofluids. In this study, three commonly models, namely Einstein [49] and Wang [50], have been decided to evaluate their accuracy in forecasting the dynamic viscosity of the studied nanofluid. The outcomes obtained from these models are then compared with the experimental data. Fig. 18 shows the comparison between the theoretical models that were chosen and the experimental data. It is notable that each of these models lacks the capability to accurately estimate the dynamic viscosity of the nanofluid. Therefore, it can be concluded that it is necessary to provide a model that has the capability to predict with precision the dynamic viscosity of the nanofluid within an acceptable range. Thus, it is required to propose a model that can accurately predict the dynamic viscosity of ZrO_2_/DW and SiC/DW mono nanofluid. Likewise, an experimental data set was compiled from the outcomes of previous researchers and is shown in Table 3 to verify the validity of the correlations in this work. This table compares different kinds of nanofluid, that had different kinds of nanoparticles, solid volume fraction, and temperature range. These results demonstrate that the proposed correlation can evaluate the experimental results of other research at different temperatures and volume solid fractions.

**Table 3:** The comparison between relative viscosity obtained by proposed correlation with experimental data obtained by other researchers.

Researchers	Nanoparticle	**Base fluid**	Temperature (^◦^C)	**Solid volume fraction (%)**	Experimental data	Calculated data
J. Fal [51]	SiO_2_	EG	25	0.02	1.34	1.38
J. Fal [51]	SiO_2_	EG	25	0.026	1.39	1.4
A. Mariano [52]	Co_3_O_4_	EG	30	0.05	1.066	1.03
M.H. Esfe [53]	ZnO	EG	40	0.012	1.017	1.21
M.H. Esfe [53]	ZnO	EG	40	0.01	1.101	1.24
Attari et al. [54]	Fe_2_O_3_	Oil	40	1	1.21	1.24
Attari et al. [54]	ZnO	Oil	40	1	1.42	1.35
Attari et al. [54]	NiO	Oil	40	1	1.91	1.24
Kole et al. [55]	Al_2_O_3_	Oil	10	0.004	1.17	1.08
Kole et al. [55]	Al2O_3_	Oil	10	0.007	1.43	1.19

### 3.9 Viscosity sensitivity

**Fig. 19:**
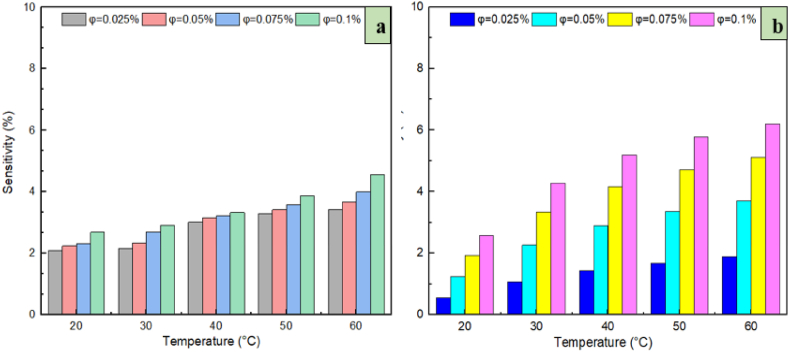
Sensitivity analysis for (a) ZrO_2_/DW and (b) SiC/DW nanofluid.

Sensitivity analysis is conducted to determine the extent to which changes in a specific model's output are caused by corresponding changes in the model's input factors (parameters or variables). For instance, when the model's parameters or input variables are changed slightly and the output is affected comparatively significantly, it is considered that the output has sensitivity to the parameters or variables. This type of analysis is generally conducted by performing a number of consecutive experiments where the designer of the model employs various input variables to identify how changing an input influences changes in the model's output. In the current study, sensitivity analysis was based on Eq. (7). Fig. 19 plots the viscosity sensitivity values against volume (***φ***) with a variation of solid volume fraction for the nanofluids of ZrO_2_/DW and SiC/DW. This indicates that the greatest change to sensitivity happened when the volume was highest, which equated to 4.55 % and 6.19 % for ZrO_2_/DW and SiC/DW, respectively. Moreover, in this figure it is shown that when the temperature remains the same and the volume fraction changes, the sensitivity increase is higher compared to when the temperature changed, and the volume remains the same. Hence, the conclusion can be made that the sensitivity of the volume fractions' objective function was greater compared to temperature, which should therefore be taken into account when preparing nanofluids, particularly when the volume fraction is 0.1 % such that an error reduction can be achieved when analysing rheological behaviour.

(7) $$\text{Sensitivity}\;\left(\%\right)=\left[\frac{{\text{RDV}}_{\text{after}\;\text{change}}}{{\text{RDV}}_{\text{before}\;\text{change}}}-1\right]\times 100$$

## 4 Conclusion

This study is aimed at determining the changes observed in dynamic viscosity of nanofluids of ZrO_2_/DW and SiC/DW with fluctuations in temperature and solid volume fraction. According to the findings of the experiments, the conclusions detailed below can be drawn.

• A rise in the temperature caused the nanofluid's viscosity to decrease. Van der Waals forces are weakened as the temperature rises. Thus, the viscosity decreased when the temperature rose.
• Newtonian behaviour was exhibited by the investigated nanofluids across the range of temperatures and solid volume fraction studied.
• The results indicate that dynamic viscosity increased to the maximum level at a solid concentration of 0.1 % and temperature of 60 °C, where the increase was around 226.3 % for the nanofluid of ZrO_2_/water and 110.5 % for the nanofluid of SiC/water.
• Through the application of fitting method to the data obtained from experiments, new correlations capable of predicting the investigated nanofluids' dynamic viscosity with respect to solid concentration and temperature has been developed.
• The comparison of the laboratory data with the outcomes from the proposed correlation showed a 5.38 % margin of deviation and R^2^ value of 98.52 % for ZrO_2_/DW and by 5.27 % and 99.11 %, respectively for SiC/DW nanofluids.

## Data availability statement

Data will be made available on request.

## CRediT authorship contribution statement

**Ahmed M. Ajeena:** Conceptualization, Data curation, Formal analysis, Investigation, Methodology, Validation, Visualization, Writing – original draft, Writing – review & editing. **Istvan Farkas:** Data curation, Formal analysis, Investigation, Supervision, Validation, Writing – review & editing. **Piroska Víg:** Conceptualization, Data curation, Formal analysis, Investigation, Resources, Supervision, Writing – review & editing.

## Declaration of competing interest

The authors declare that they have no known competing financial interests or personal relationships that could have appeared to influence the work reported in this paper.

## References

[1] Sajid M.U., Ali H.M. (2019). Recent advances in application of nanofluids in heat transfer devices: a critical review. Renew. Sustain. Energy Rev..

[2] AbuShanab Y., Al-Ammari W.A., Gowid S., Sleiti A.K. (2023). Accurate prediction of dynamic viscosity of polyalpha-olefin boron nitride nanofluids using machine learning. Heliyon.

[3] Ajeena A.M., Víg P., Farkas I. (2022). A comprehensive analysis of nanofluids and their practical applications for flat plate solar collectors: fundamentals, thermophysical properties, stability, and difficulties. Energy Rep..

[4] Ganesh Kumar P., Sakthivadivel D., Meikandan M., Vigneswaran V.S., Velraj R. (2019). Experimental study on thermal properties and electrical conductivity of stabilized H2O-solar glycol mixture based multi-walled carbon nanotube nanofluids: developing a new correlation. Heliyon.

[5] Hemmat Esfe M., Motallebi S.M., Toghraie D. (2022). Optimal viscosity modelling of 10W40 oil-based MWCNT (40%)-TiO2 (60%) nanofluid using Response Surface Methodology (RSM). Heliyon.

[6] Assael M.J., Antoniadis K.D., Wakeham W.A., Zhang X. (2019). Potential applications of nanofluids for heat transfer. Int. J. Heat Mass Tran..

[7] Esfe M.H., Toghraie D., Esfandeh S., Alidoust S. (2022).

[8] Mukesh Kumar P.C., Kavitha R. (2020). Regression analysis for thermal properties of Al2O3/H2O nanofluid using machine learning techniques. Heliyon.

[9] Syam Sundar L., Venkata Ramana E., Singh M.K., De Sousa A.C.M. (2012). Viscosity of low volume concentrations of magnetic Fe 3O 4 nanoparticles dispersed in ethylene glycol and water mixture. Chem. Phys. Lett..

[10] Yu W., Xie H., Chen L., Li Y. (2009). Investigation of thermal conductivity and viscosity of ethylene glycol based ZnO nanofluid. Thermochim. Acta.

[11] Khedkar R.S., Shrivastava N., Sonawane S.S., Wasewar K.L. (2016). Experimental investigations and theoretical determination of thermal conductivity and viscosity of TiO2-ethylene glycol nanofluid. Int. Commun. Heat Mass Transf..

[12] Baratpour M., Karimipour A., Afrand M., Wongwises S. (2016). Effects of temperature and concentration on the viscosity of nanofluids made of single-wall carbon nanotubes in ethylene glycol. Int. Commun. Heat Mass Transf..

[13] Elias M.M., Mahbubul I.M., Saidur R., Sohel M.R., Shahrul I.M., Khaleduzzaman S.S., Sadeghipour S. (2014). Experimental investigation on the thermo-physical properties of Al2O3 nanoparticles suspended in car radiator coolant. Int. Commun. Heat Mass Transf.

[14] Said Z., Sajid M.H., Alim M.A., Saidur R., Rahim N.A. (2013). Experimental investigation of the thermophysical properties of AL2O3-nanofluid and its effect on a flat plate solar collector. Int. Commun. Heat Mass Transf..

[15] Hemmat Esfe M., Saedodin S., Mahmoodi M. (2014). Experimental studies on the convective heat transfer performance and thermophysical properties of MgO-water nanofluid under turbulent flow. Exp. Therm. Fluid Sci..

[16] Hemmat Esfe M., Saedodin S., Mahian O., Wongwises S. (2014). Thermophysical properties, heat transfer and pressure drop of COOH-functionalized multi walled carbon nanotubes/water nanofluids. Int. Commun. Heat Mass Transf..

[17] Duan F., Wong T.F., Crivoi A. (2012). Dynamic viscosity measurement in non-Newtonian graphite nanofluids. Nanoscale Res. Lett..

[18] Tseng W.J., Lin K.C. (2003). Rheology and colloidal structure of aqueous TiO2 nanoparticle suspensions. Mater. Sci. Eng. A..

[19] Kulkarni D.P., Das D.K., Chukwu G.A. (2006). Temperature dependent rheological property of copper oxide nanoparticles suspension (nanofluid). J. Nanosci. Nanotechnol..

[20] Wang L., Wang Y., Yan X., Wang X., Feng B. (2016). Investigation on viscosity of Fe3O4 nanofluid under magnetic field. Int. Commun. Heat Mass Transf..

[21] Sabiha M.A., Mostafizur R.M., Saidur R., Mekhilef S. (2016). Experimental investigation on thermo physical properties of single walled carbon nanotube nanofluids. Int. J. Heat Mass Tran..

[22] Choi S.U.S. (1995). Enhancing thermal conductivity of fluids with nanoparticles. Am. Soc. Mech. Eng. Fluids Eng. Div. FED..

[23] Saeedi A.H., Akbari M., Toghraie D. (2018). An experimental study on rheological behavior of a nanofluid containing oxide nanoparticle and proposing a new correlation. Phys. E Low-Dimensional Syst. Nanostructures..

[24] Hemmat Esfe M., Mohammadnejad Ardeshiri E., Toghraie D. (2022). Application of experimental and statistical methods in the study of rheology of MWCNT (25%)-TiO2 (75%)/SAE40 HNF to identify and use in the lubrication industry. Colloids Surfaces A Physicochem. Eng. Asp..

[25] Li X., Wang H., Luo B. (2021). The thermophysical properties and enhanced heat transfer performance of SiC-MWCNTs hybrid nanofluids for car radiator system. Colloids Surfaces A Physicochem. Eng. Asp..

[26] Bahrami M., Akbari M., Karimipour A., Afrand M. (2016). An experimental study on rheological behavior of hybrid nanofluids made of iron and copper oxide in a binary mixture of water and ethylene glycol: non-Newtonian behavior. Exp. Therm. Fluid Sci..

[27] Toghraie D., Alempour S.M., Afrand M. (2016). Experimental determination of viscosity of water based magnetite nanofluid for application in heating and cooling systems. J. Magn. Magn Mater..

[28] Etaig S., Hasan R., Perera N. (2016). Investigation of a new effective viscosity model for nanofluids. Procedia Eng..

[29] Hemmat Esfe M., Motallebi S.M., Toghraie D. (2022). Investigation of thermophysical properties of MWCNT-MgO (1:1)/10W40 hybrid nanofluid by focusing on the rheological behavior: sensitivity analysis and price-performance investigation. Powder Technol..

[30] Afrand M., Toghraie D., Ruhani B. (2016). Effects of temperature and nanoparticles concentration on rheological behavior of Fe3O4-Ag/EG hybrid nanofluid: an experimental study. Exp. Therm. Fluid Sci..

[31] Pastoriza-Gallego M.J., Casanova C., Legido J.L., Piñeiro M.M. (2011). CuO in water nanofluid: influence of particle size and polydispersity on volumetric behaviour and viscosity. Fluid Phase Equilib.

[32] Azmi W.H., Sharma K.V., Sarma P.K., Mamat R., Anuar S., Dharma Rao V. (2013). Experimental determination of turbulent forced convection heat transfer and friction factor with SiO2 nanofluid. Exp. Therm. Fluid Sci..

[33] Duangthongsuk W., Wongwises S. (2009). Measurement of temperature-dependent thermal conductivity and viscosity of TiO2-water nanofluids. Exp. Therm. Fluid Sci..

[34] Hojjat M., Etemad S.G., Bagheri R., Thibault J. (2011). Rheological characteristics of non-Newtonian nanofluids: experimental investigation. Int. Commun. Heat Mass Transf..

[35] Phuoc T.X., Massoudi M., Chen R.H. (2011). Viscosity and thermal conductivity of nanofluids containing multi-walled carbon nanotubes stabilized by chitosan. Int. J. Therm. Sci..

[36] Rudyak V.Y., Dimov S.V., Kuznetsov V.V., Bardakhanov S.P. (2013). Measurement of the viscosity coefficient of an ethylene glycol-based nanofluid with silicon-dioxide particles. Dokl. Phys..

[37] Sadri R., Ahmadi G., Togun H., Dahari M., Kazi S.N., Sadeghinezhad E., Zubir N. (2014). An experimental study on thermal conductivity and viscosity of nanofluids containing carbon nanotubes. Nanoscale Res. Lett..

[38] Li H., Wang L., He Y., Hu Y., Zhu J., Jiang B. (2014). Experimental investigation of thermal conductivity and viscosity of ethylene glycol based ZnO nanofluids. Appl. Therm. Eng..

[39] Jarahnejad M., Haghighi E.B., Saleemi M., Nikkam N., Khodabandeh R., Palm B., Toprak M.S., Muhammed M. (2015). Experimental investigation on viscosity of water-based Al2O3 and TiO2 nanofluids. Rheol. Acta.

[40] Abbasi S., Zebarjad S.M., Baghban S.H.N., Youssefi A., Ekrami-Kakhki M.S. (2016). Experimental investigation of the rheological behavior and viscosity of decorated multi-walled carbon nanotubes with TiO2 nanoparticles/water nanofluids. J. Therm. Anal. Calorim..

[41] Meybodi M.K., Daryasafar A., Koochi M.M., Moghadasi J., Meybodi R.B., Ghahfarokhi A.K. (2016). A novel correlation approach for viscosity prediction of water based nanofluids of Al2O3, TiO2, SiO2 and CuO. J. Taiwan Inst. Chem. Eng..

[42] Hemmat Esfe M. (2018). The investigation of effects of temperature and nanoparticles volume fraction on the viscosity of Copper Oxide-ethylene Glycol Nanofluids. Period. Polytech. - Chem. Eng..

[43] Murshed S.M.S., Leong K.C., Yang C. (2008). Investigations of thermal conductivity and viscosity of nanofluids. Int. J. Therm. Sci..

[44] Chandrasekar M., Suresh S., Chandra Bose A. (2010). Experimental investigations and theoretical determination of thermal conductivity and viscosity of Al2O3/water nanofluid. Exp. Therm. Fluid Sci..

[45] Yiamsawas T., Dalkilic A.S., Mahian O., Wongwises S. (2013). Measurement and correlation of the viscosity of water-based Al2O3 and TiO2 nanofluids in high temperatures and comparisons with literature reports. J. Dispers. Sci. Technol..

[46] Jeong J., Li C., Kwon Y., Lee J., Kim S.H., Yun R. (2013). Particle shape effect on the viscosity and thermal conductivity of ZnO nanofluids. Int. J. Refrig..

[47] (2009). A., Handbook, ASHRAE Handbook: Fundamentals.

[48] Venerus D.C., Buongiorno J., Christianson R., Townsend J., Bang I.C., Chen G., Chung S.J., Chyu M., Chen H., Ding Y., Dubois F., Dzido G., Funfschilling D., Galand Q., Gao J., Hong H., Horton M., Hu L., Iorio C.S., Jarzebski A.B., Jiang Y., Kabelac S., Kedzierski M.A., Kim C., Kim J.H., Kim S., McKrell T., Ni R., Philip J., Prabhat N., Song P., Van Vaerenbergh S., Wen D., Witharana S., Zhao X.Z., Zhou S.Q. (2010). Viscosity measurements on colloidal dispersions (nanofluids) for heat transfer applications. Appl. Rheol..

[49] Einstein A. (1906). Eine neue bestimmung der Moleküldimensionen. Ann. Phys..

[50] Wang X., X X. (1999). Thermal conductivity of nanoparticle–fluid mixture. J. Thermophys. Heat Transf..

[51] Fal J. (2017). Thermochimica Acta Viscosity , thermal and electrical conductivity of silicon dioxide – ethylene glycol transparent nanofluids. An experimental studies.

[52] Mariano A., Pastoriza-gallego M.J., Lugo L., Mussari L., Piñeiro M.M. (2015). International Journal of Heat and Mass Transfer Co 3 O 4 ethylene glycol-based nanofluids : thermal conductivity , viscosity and high pressure density. Int. J. Heat Mass Tran..

[53] Esfe M.H., Saedodin S. (2014). An experimental investigation and new correlation of viscosity of ZnO – EG nanofluid at various temperatures and different solid volume fractions. Exp. Therm. Fluid Sci..

[54] Attari H., Derakhshanfard F., Hossein M., Darvanjooghi K. (2017). Effect of temperature and mass fraction on viscosity of crude oil-based nano fl uids containing oxide nanoparticles. Int. Commun. Heat Mass Transf..

[55] Namburu P.K., Das D.K., Tanguturi K.M., Vajjha R.S. (2009). Numerical study of turbulent flow and heat transfer characteristics of nanofluids considering variable properties. Int. J. Therm. Sci..

